# Impact of the tripartite interaction between rice, sheath blight and diverse crop-associated endophytes on phenotypic and biochemical responses in rice

**DOI:** 10.1016/j.heliyon.2024.e32574

**Published:** 2024-06-06

**Authors:** Aditya Kukreti, Chethana Bangi Siddabasappa, Prasannakumar Muthakapalli Krishnareddy, Yashavanth Basavapatna Subbanna, Manjunatha Channappa, Shivakumara Kadanakuppe Thammayya, Eman A. Mahmoud, Rafa Almeer

**Affiliations:** a Department of Plant Pathology, University of Agricultural Sciences, GKVK, Bengaluru, 560 065, India; b National Academy of Agricultural Research Management, Hyderabad, Telangana, 500 030, India; c Insect Bacteriology Laboratory, ICAR-National Bureau of Agricultural Insect Resources, Bengaluru, 560 024, India; d Department of Food Sciences, College Agriculture, Damietta University, 34511, Egypt; e Department of Zoology, College of Science, King Saud University, 11451, Saudi Arabia

**Keywords:** Bacterial endophytes, Plant growth promotion, Polyphenol oxidase, Peroxidase, Superoxide dismutase, And phenylalanine ammonia lyase

## Abstract

Endophytes stimulate plant growth and inhibit phytopathogens. Most of the known endophytes are host-specific and only a few strains are effective for practical field use. Thus, this study focuses on the evaluation of endophytes *viz*., *Bacillus pseudomycoides* strain HP3d, *Paenibacillus polymyxa* strain PGSS1, *B. velezensis* strain A6 and P42 isolated from diverse crop ecosystems for their potential to promote plant growth and induce systemic resistance against sheath blight disease in rice. The endophytes were studied for plant growth promoting traits *in vivo* conditions and were found to exhibit ammonia (light to strong), siderophore (yellow zone on the CAS agar plate), indole-3-acetic acid (15.20–22.19 μg mL^−1^) production and phosphorus solubilization (1.2–1.5 cm). In the glasshouse, when applied individually and in combinations through various methods like seed treatment, seedling dip, and foliar spray these endophytes significantly reduced lesion size (2.06–2.37 fold) and ShB severity (2.60–2.58 fold), enhancing growth parameters *viz*., shoot (1.09–1.11 fold), root (1.02–1.20 fold), number of tillers (1.2–1.6 fold), shoot (80.58–82.64 %) and root (62.01–66.66 %) dry matter over untreated control. Consequently, enzyme activity *viz*., polyphenol oxidase (2.20–3.00 U^−1^min^−1^g^−1^), peroxidase (0.31–0.35 min^−1^g^−1^), superoxide dismutase (118.50–123.00 Ug^−1^ FW), and phenylalanine ammonia lyase (0.84–0.90 min⁻^1^g⁻^1^FW) was found to increase up to the fourth day after the pathogen challenge and subsequently decrease thereafter. Chlorophyll content post inoculation of ShB declined over time but endophyte treated plants exhibited lesser reductions over uninoculated control. Field trials corroborated the *in vitro* findings, demonstrating reduced ShB (1.71–1.88 fold decrease in PDI) and enhanced growth (1.1–1.2 fold increase in shoot length) over untreated controls. The combined application of seedling dip, seed treatment, and foliar spray proved to be the most optimum treatment. The findings highlight the potential of diverse crop-derived endophytes, emphasizing their non-host specificity and effectiveness as broad-spectrum bioagents in actual field conditions.

## 1 Introduction

Endophytes are microorganisms that live inside the host plants and positively affect plant growth and development. Many bacterial and fungal endophytes are known to have plant growth-promoting (PGP) ability and act as efficient biocontrol agents (BCAs). Within endophytes, bacteria are regarded as the most efficient BCAs due to their quick development, simplicity of handling and aggressive colonizing characteristics [1] and among bacteria, *Bacillus* spp. stands out as a promising biocontrol agent due to its Gram-positive nature, ability to produce endospores, and resistance to heat and desiccation. These characteristics enhance its storage stability and effectiveness in field applications [2]. Numerous *Bacillus* spp. have been recorded for their antagonistic activity against plant pathogens, with *B. velezensis* being extensively studied in recent years for its biocontrol potential whereas *B. pseudomycoides*, a novel species, is still unexplored [[3], [4], [5], [6]]. Apart from *Bacillus* sp., *Paenibacillus* sp. are well-known endophytes, that release many different hydrolyzing enzymes that aid in the colonization of plant tissue [7].

The world's most consumed cereal crop, rice is imperative for the fast-expanding populations in South Asian nations and contributes 20 % of the protein in the diets of emerging nations [8]. Several phytopathogens reduce rice yield and result in significant economic loss annually. Among them, sheath blight (*Rhizoctonia solani* Kuhn) stands as among the most detrimental fungal diseases globally and is a significant production barrier, resulting in annual yield losses of up to 10 %–30 % that could increase to 50 % in the coming years [9]. Due to the widespread cultivation of high-yielding rice varieties and hybrids with limited genetic diversity, extensive reliance on chemical fertilizers, and due to changes in the environment, it is difficult to control sheath blight [10]. Farmers primarily use fungicides to manage this pathogen, but their adverse environmental effects and low cost-benefit ratio have led to employing biological control methods that offer a safer alternative for decreasing the reliance on harmful chemicals [11]. Thus, under the Integrated Pest Management (IPM) framework, BCAs like *B. velezensis*, *B. pseudomycoides* and *P. polymyxa* can offer efficient and sustainable means to manage sheath blight.

Our previous studies identified copious bioactive secondary metabolites like antimicrobial peptides of *B. velezensis* strains A6 and P42 and found these isolates as potential biocontrol agents [12,13]. Apart from secondary metabolite production, endophytes generally stimulate induced systemic resistance (ISR) *via* activation of enzymes of phenylpropanoid pathway such as peroxidase, superoxide dismutase, polyphenol oxidase and phenylalanine ammonia-lyase [14,15] in plants, as biocontrol mechanisms. They also stimulate plant growth by aiding nutrient acquisition and modulating phytohormone levels [16,17]. Consequently, plant-beneficial endophytic bacteria have great potential to be used as biofertilizers and biopesticides. However, many known strains are generally ineffective under field conditions [18]. To the best of our knowledge, there are no known studies on the antagonistic ability of the non-host endophytes *i.e.*, *B. velezensis*, *P. polymyxa* and *B. pseudomycoides*, isolated from diverse crops, against sheath blight of rice, as endophyte host specificity has been the subject of conflicting reports. According to several studies, endophytes can only encourage plant growth related to their original host. Endophytes, on the other hand, have been linked to the growth of a variety of plant hosts [19]. Also, there are lacunae in studies regarding these endophytes been compared *in vitro* and *in planta* within the rice ecosystem.

In view of the above information, this study aims to examine characterized bacterial endophytic strains, *B. velezensis* strains A6 and P42, *P. polymyxa* PGSS-1, and *B. pseudomycoides* HP3d for various PGP attributes, including ammonia production, siderophore production, IAA production, and phosphorus solubilization *in vitro*. Additionally, the biocontrol capabilities of these strains were assessed by enzyme assays measuring polyphenol oxidase, peroxidase, phenyl ammonia lyase, and superoxide dismutase activities. The effectiveness of these strains in promoting plant growth and controlling sheath blight disease was further studied in both glasshouse and field conditions by measuring different morphological parameters of rice.

## 2 Materials and method

### 2.1 Procurement of bacterial strains, plant material and pathogen isolation

The characterized non-pathogenic four endophytic bacterial strains (*Bacillus velezenesis* strain P42 [from pomegranate, accession number KC692168.3]; *B. velezenesis* strain A6 [from rice, accession number MSXZ01000332.1]; *Paenibacillus polymyxa* strain PGSS-1 [from ragi] *B. pseudomycoides* strain HP3d [from rice, accession number MH465502]) were procured from Bacteriology Laboratory, Department of Plant Pathology, UAS, Bengaluru, Karnataka, India and were preserved at 4 °C and stored at −20 °C with 20 % glycerol for further study. The rice variety Jyothi (susceptible to sheath blight) was used to assess the efficacy of endophytes against sheath blight disease in rice. *Rhizoctonia solani* was isolated from infected leaf sheath samples by the tissue segment method [20] on PDA media. The pathogenicity of *R. solani* was proved following Koch's postulates.

### 2.2 *In vitro* assessment of endophytes for plant growth promoting (PGP) attributes

Different assays were performed to assess the PGP attributes of four endophytes. Ammonia production was evaluated using the method of Cappuccino and Sherman, 1992 [21], using the culture grown in buffered peptone water for 96 h at 30 ± 0.1 °C. In the phosphate solubilization assay, endophytes underwent screening to evaluate their capacity for solubilizing Tricalcium phosphate (TCP) as insoluble inorganic phosphate sources on Pikovskaya's (PVK) agar medium [22]. A qualitative assessment of the siderophore-producing ability of bacterial strains was conducted through the Universal CAS-Agar Plate assay [23]. The IAA (Indole-3-acetic acid) was analyzed using qualitative and quantitative methods. For qualitative assay, the phytohormone IAA being an indole compound was detected by following the method delineated by Gordon and Weber (1951), whereas for quantitative assay, the standard curve was constructed by using different concentrations of IAA *viz.,* 5, 10, 20, 30 and 40 μg mL^−1^ [24]. Indole-3-acetic acid (IAA) concentration was determined in the culture filtrate by substituting the OD value as the Y value in the slope formula and determining the X value i.e., IAA concentration.

### 2.3 Evaluation of endophytes for their plant growth-promoting (PGP) activity in rice under glasshouse conditions

#### 2.3.1 Effect of endophytes on plant growth

The four endophytes and *P. fluorescence* (positive control) grown in Luria Bertani broth for 48 h at 28 ± 2 °C were centrifuged (6000×*g* for 5 min), washed and resuspended in sterile water (suspension of 10^9^ cfu/mL). PGP activity for four endophytes and *P. fluorescence* was assessed in a glasshouse with six modes of application and three replicates each. The treatments included seed treatment (ST) with endophytes (T1), seedling dip (SD) with endophytes (T2), ST combined with foliar spray (FS) with endophytes (T3), SD combined with FS with endophytes (T4), ST in conjunction with SD with endophytes (T5), and combination of ST, SD and FS with endophytes (T6). The rice seeds were segregated into two segments A and B, the first portion A was further divided based on bacterization with five endophytes. For only SD treatment and for negative control, non-inoculated, surface-sterilized portion B was employed.

To carry out surface sterilization for ST (treatment T1), segment A rice seeds underwent treatment with NaClO for 2 min, followed by thorough rinsing thrice with sterile water. Subsequently, seeds underwent treatment with 10 mL/kg (10^9^ cfu/mL) fresh endophytic cultures (48 h) grown in nutrient broth amended with 0.2 % CMC @ 5 mL/g of seed (act as a sticker). After seed treatment, the seeds were incubated for 24 h and then shade dried. Subsequently, the treated seeds were sown in sterile soil in plastic trays and were kept in the glasshouse for 21 days. Uninoculated seeds served as the negative control, while seeds treated with *P. fluorescence* were designated as the positive control. For SD (treatment T2), pots sterilized and were filled with soil: cocopeat: FYM in a 1:1:1 ratio. The field soil and cocopeat were autoclaved twice before filling for 45 min at 121 °C with 24 h intervals. A section of portion B seeds (uninoculated) was selected, planted, and subsequently transferred into half of these pots 21 days after sowing (DAS) after dipping in 48 h old endophyte culture (diluted in water @ 10 mL/L) for 30 min. The remaining half of the pots received transplantation with a fraction of the seedlings from portion A, which were dipped in a 48-h-old endophyte culture constituting treatment T5.

FS of 24 h old endophytes suspension (1 × 10^9^ CFU/mL) diluted in water @ 10 mL/L was done for part of ST (treatment T3), SD (treatment T4), and ST + SD (treatment T6) seedlings, 10 days after transplanting (DAT). The parameters *viz.*, stem height, root length and number of tillers for rice plants were recorded at 20 DAT. The weight of roots and shoots, both fresh and dry, was also recorded post-harvest by drying to a constant weight in an oven at 60 °C for 3 days.

#### 2.3.2 Effect of endophytes on lesion size and sheath blight intensity

To study the antagonism of endophytes against sheath blight pathogen, the 35 DAS (days after sown) plants in the above six treatments were challenge inoculated with *R. solani* (by carefully opening the leaf sheaths of plants positioned 2 cm above the water level, inoculating them with sclerotia at a rate of one sclerotium per plant, and subsequently covering the treated area with aluminium foil). Symptom progression was noted five days post-inoculation and after the emergence of characteristic lesions within five days, the aluminum foil was taken off, and the affected plants were placed within a humidity chamber to facilitate normal disease progression. The chamber maintained ideal humidity levels of 90 ± 3 % and a consistent temperature of 30° ± 4 °C. The plants remained in the humidity chamber for 14 days to support the typical development of the disease. Plants without inoculation were maintained as the negative control. Symptoms were graded based on lesion size (measured by a ruler) and severity of sheath blight was calculated by dividing the lesion height by plant height and expressed in percentage after 2 weeks of inoculation *i.e*., 49 days old plant [15].

### 2.4 Sample collection, enzyme assays and chlorophyll estimation

For studying enzymatic activities within the above glasshouse setup, sampling was conducted by taking matured leaves on the 0^th^, 2nd, 3rd, 4th and 5th days of challenge inoculation with *R. solani*. One gm of leaf sample was homogenization using liquid nitrogen with a pre-cooled pestle and mortar and the resulting powder was grinded in a 3 mL solution comprising 0.1 M sodium phosphate buffer with polyvinylpyrrolidone (PVP) and ethylenediaminetetraacetic acid (EDTA) at pH 7.0 [14,25]. The homogenate was centrifuged at 10,000 rpm for 20 min at 4 °C and the obtained supernatant served as a crude enzyme extract for subsequent assay *viz*., activities of polyphenol oxidase, peroxidase, phenyl ammonia lyase, and superoxide dismutase. All the steps in extract preparation were carried out at 4 °C**.** Each enzyme assay consisted of three replications (each replication consists of five plants) and two spectrophotometric readings per replication.

#### 2.4.1 Polyphenol oxidase (PPO) activity

The PPO activity was assessed following the methodology of Manoranjan and Mishra (1976). The change in absorbance was measured at 420 nm at intervals of 30 s for 3 min from zero second of incubation. The result was indicated as a change in absorbance/min/g of fresh tissue [26].

#### 2.4.2 Peroxidase (POD) activity

An assay of POD activity was carried out as detailed by Saroop et al. (2002). The increase in absorbance at 420 nm was monitored for 3 min at 30 s intervals for the measurement of activity and was indicated as a change in absorbance/min/g of fresh tissue [27].

#### 2.4.3 Superoxide dismutase (SOD) activity

The assay for SOD was conducted based on its ability to inhibit the nitro blue tetrazolium (NBT), photochemical reduction following Beauchamp and Fridovich (1971). The activity SOD enzyme was indicated in units g ^−1^ fresh weight [28].

#### 2.4.4 Phenylalanine ammonia lyase (PAL) activity

PAL activity was assayed by Campos et al. (2004). The assay mixture was read at 290 nm and was articulated as a change in absorbance/min/g of fresh tissue [29].

### 2.5 Chlorophyll estimation

Chlorophyll content assessment was performed on the 0^th^, 3rd and 5th days following the challenge inoculation with *R. solani*, according to the protocol outlined by Hiscox and Israelstan (1979) [30]. Chlorophyll *a*, *b* and total chlorophyll content were computed through the following formulas [31].

$$\text{Chlorophyll}\;‘a’=\left(12.7\;\times \;A_{663}\;-\;2.69\times A_{645}\right)\;\times \;\frac{V}{1000}\;\times \;\frac{1}{\text{Fresh}\;\text{weight}\;\left(\text{mg}/g\right)}$$

$$\text{Chlorophyll}\;‘b’=\left(22.9\times \;A_{645}\;–\;4.68\;{\text{×A}}_{663}\right)\;\times \;\frac{V}{1000}\;\times \;\frac{1}{\text{Fresh}\;\text{weight}\;\left(\text{mg}/g\right)}$$

$$\text{Total}\;\text{chlorophyll}=\left(20.2\;X\;A_{645}+8.02\;x\;A_{663}\right)\;\times \;\frac{V}{1000}\;\times \;\frac{1}{\text{Fresh}\;\text{weight}\;\left(\text{mg}/g\right)}$$

where, A645 = absorbance of extract at 645 nm, A663 = absorbance of extract at 663 nm,

V = volume of the extract (10 mL), and W = fresh weight of the sample (0.5 g).

### 2.6 Field evaluation of bacterial endophytes against sheath blight

Four endophytes including *P*. *fluorescence* as a positive control, uninoculated plants as negative control and a recommended fungicide, were evaluated against *R. solani* at a sick plot maintained in C block, ZARS, VC Farm, Mandya, Karnataka, India during the summer season of 2021. The experiment followed an RBD with 22 treatments, each with three replications. The 22 treatments involving endophytes were applied in the following manners: P42 treatments included ST (T1), SD (T2), a combination of ST and SD (T3), and a comprehensive approach involving ST, SD and FS (T4), all followed by challenge inoculation (CI); similarly, HP3d treatments comprised ST (T5), SD (T6), a combination of ST and SD (T7), and combination of ST, SD and FS (T8), all followed by challenge inoculation (CI); PGSS 1 treatments involved ST (T9), SD (T10), a combination of ST and SD (T11), and a comprehensive approach involving ST, SD and FS (T12), all followed by challenge inoculation (CI); A6 treatments included ST (T13), SD (T14), a combination of ST and SD (T15), and a comprehensive approach involving ST, SD and FS (T16), all followed by challenge inoculation (CI); PF (*P. fluorescence*) treatments consisted of ST (T17), SD (T18), a combination of ST and SD (T19), and a comprehensive approach involving ST, SD and FS (T20), all followed by challenge inoculation (CI). Additionally, the study included a fungicide Hexaconazole 5SC at 2 mL/L in rice (T21) and negative control with no treatment (T22).

The rice seedlings were transferred into the experimental plots measuring 5 × 2 m^2^, with a spacing of 15 × 10 cm. The recommended agricultural practices such as irrigation and fertilization were applied timely in the field. Different methods of application of endophytes followed in field conditions were ST, SD, FS, and their combinations. For ST, rice seeds underwent surface sterilization by immersion in a 2 % NaClO solution for 2 min, followed by thorough rinsing thrice in sterile distilled water to eliminate any remaining traces. The seeds were air-dried under a sterile air stream. Further, these seeds were immersed in fresh culture broth of endophytes @ 10 mL/kg of seed along with Tween 20 @10 mL/L of H_2_O. (amended with 0.2 % CMC at 5 mL/g) having 1 × 10^9^ CFU/mL concentration; air dried and were sown in nursery. For SD, the seedlings of rice (30 DAS) were dipped in the fresh culture broth of endophytes (amended with 0.2 % CMC @ 5 mL/g) with CFU 1 × 10^9^/mL diluted @ 10 mL/L of H_2_O before transplanting into the field. FS was carried out by spraying fresh endophyte culture diluted @10 mL/L along with Tween 20 (0.1 %) to plants at 15 days interval after the initiation of symptoms during cropping season. A total of three sprays were given.

Observations on per cent incidence of sheath blight, growth parameters, plant height and the number of tillers were recorded at 80 DAT and grain yield/ha after harvesting was also recorded. Disease scoring after each treatment spray was made following the 0–9 Standard Evaluation System 2013 (IRRI, 2013). From each plot 10 plants were taken randomly and the PDI (percent disease index) and PDOC (percent disease decreased over control) were calculated.

### 2.7 Statistical analysis

The normality of error distribution was assessed using the Shapiro-Wilk test, while the homogeneity of error variance was investigated through the Bartlett test [32]. In instances where the assumptions of normality and homogeneity of variance were satisfied, an ANOVA followed by the Tukey test was employed to compare all treatments, with a significance level set at 5 %. Data was analyzed and subjected to two-way ANOVA in the case of the glasshouse, one-way ANOVA in the case of field conditions and the least significant difference test (LSD) was performed to separate the group means when ANOVAs were significant at P < 0.05. Before statistical analysis, all percentage data underwent an angular transformation to stabilize variances. The means were compared by Tukey's Honest Significance Difference (HSD). Graphs were generated using the package ggplot2 in R software.

## 3 Results

### 3.1 *In vitro* assessment of endophytes for plant growth promoting (PGP) attributes

**Fig. 1:**
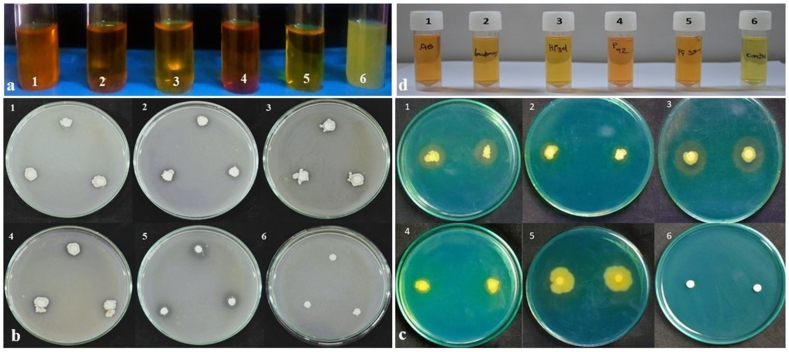

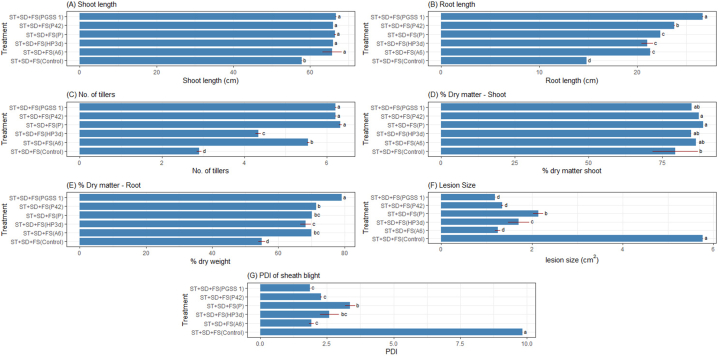
(1A) Comparative analysis of (a) ammonia production, (b) phosphorus solubilization, (c) siderophore production (d) IAA production, by bacterial endophytes (1) *P. fluorescence*, (2) *B. velezensis* P42, (3) *B. pseudomycoides* HP3d, (4) *P. polymyxa* PGSS1, (5) *B. velezensis* A6, and (6) control. (**1B)** Effect of ST + SD + FS of bacterial endophytes on rice under glasshouse conditions. Bar graph represents (A) Shoot length (B) Root length, (C) No. of tillers (D) % Dry matter shoot (E) % Dry matter root (F) Lesion size (cm^2^) and (G) PDI of sheath blight. The figures presented are the mean values of three independent experiments. Standard errors of the mean values are presented as bars. Letters on bars indicate DMRT at *p* < 0.05.

All four endophytes exhibited positive results for ammonia production (Fig. 1A(a)). The development of brown colour of the culture with the addition of Nessler reagent (1 mL, 70.83 g/L K₂HgI₄ in 2.5 M KOH) indicated the ammonia production. The qualitative result was derived by the production of dark brown, light-yellow and no colour which indicates strong, light and no ammonia production respectively. *B. velezenesis* strain P42 and *P. polymyxa* PGSS1 showed strong ammonia production whereas light ammonia production was seen in *B. velezenesis* strain A6 and *P. fluorescence*. No alteration in colour was noted for the control. Qualitative and quantitative assay of endophytic bacterial isolates regarding phosphate solubilization exhibited the ability of all four bacterial endophytes to solubilize phosphorus in the plate-based assay, as evidenced by a clear halo around the colony (Fig. 1A(b)). The endophytes i.e., strains A6, PGSS1 and HP3d recorded a zone of clearance of 0.2 cm while it was 0.1 cm in strain P42. The highest zone of clearance of 0.5 cm was recorded in *P. fluorescence*. Based on colony and halo diameter, the phosphorus solubilization index was 1.2 cm,1.2 cm, 1.2 cm,1.1 cm and 1.5 cm for HP3d, PGSS 1, A6, P42 and *P. fluorescens* respectively. No halo formation was recorded in the control.

The four endophytes were recorded positive for siderophore production, showing a yellow zone on the CAS agar medium plate (Fig. 1A(c)). Both qualitative and quantitative assessments were performed to assess the production of IAA by the isolates. All bacterial endophytes produced a significant amount of IAA ranging from 16.02 to 22.19 μg mL^−1^. Strain A6 recorded a maximum IAA production of 22.19 μg mL^−1^ whereas a minimum of 16.02 μg mL^−1^ by strain HP3d. All endophytes recorded high IAA production over *P. fluorescence* (15.20 μg mL^−1^) (Fig. 1A(d)).

### 3.2 Evaluation of endophytes for their plant growth-promoting (PGP) activity in rice under glasshouse conditions

#### 3.2.1 Effect of endophytes on plant growth

A two-way ANOVA test was done to assess the effect of two independent variables (endophytes and mode of treatment) on dependent variables *i.e.*, growth parameters. Bacterial endophytes effectively promoted rice growth as evidenced by significantly increased root length, number of leaves, shoot length, per cent shoot and root dry matter compared to the negative control. For shoot length, treatment by ST + SD + FS and SD + FS recorded the highest mean shoot length of 64.93 and 64.09 cm respectively, whereas ST recorded the lowest of 57.58 cm. Plants inoculated with endophytes exhibited markedly elevated level of shoot length compared to the ones inoculated with *P. fluorescence* (positive control). Interaction studies (different endophytes and different modes of treatment combinations) revealed that combination ST + SD + FS and SD + FS in all four endophyte strains and *P. fluorescence* recorded increased shoot length (61.11–66.67 cm) significant over negative control (57.89 cm) (Supplementary Table 1; Fig. 1B). In the case of root length, plants with ST + SD + FS recorded the highest mean root length of 21.66 cm whereas with ST recorded the lowest of 12.85 cm. Inoculation with PGSS1, P42 and A6 recorded the significantly highest mean root length of 17.78, 17.50, and 17.50 cm respectively thus exhibiting a 1.2-fold increase over negative control (14.83 cm), whereas HP3d recorded the lowest of 15.19 cm. *P. fluorescence* recorded the highest mean root length of 18.64 cm over all the endophytes (Supplementary Table 2). For the number of tillers, treatment with ST + SD + FS recorded the highest mean number of tillers of 5.26 (Fig. 1B), whereas ST recorded the lowest of 2.98. Strain A6 and PGSS1 recorded the significantly highest mean number of tillers 4.58 and 4.56 over positive control, *P. fluorescence* (4.30) thus exhibiting a 1.6-fold increase over negative control (2.87) (Supplementary Table 3).

The treatment with ST + SD + FS recorded the highest mean root dry matter of 69 % (Fig. 1B) whereas ST recorded the lowest of 53.36 %. Plants inoculated with strain P42 recorded the highest mean root dry matter of 66.66 % which was significantly higher compared to the ones inoculated with *P. fluorescence* (61.74 %) and negative control (54.67 %) (Supplementary Table 4). Regarding shoot dry matter, ST + SD + FS in rice recorded the highest mean of 85.25 % (Fig. 1B), whereas ST recorded the lowest of 76.64 %. Treatment with strains A6, PGSS1 and P42 recorded the highest shoot dry matter of 82.64 %, 82.02 % and 80.95 % respectively whereas with HP3d recorded the lowest of 80.58 %. All endophytes recorded significantly higher mean shoot dry weight over positive control (*P. fluorescence*, 80.07 %) and negative control (79.73 %) (Supplementary Table 5).

#### 3.2.2 Effect of endophytes on lesion size and sheath blight intensity

An ANOVA with two independent variables, endophytes, and treatment mode, was conducted to assess their combined impact on both lesion size and sheath blight intensity. Among modes of treatments, the ST + SD + FS treatment recorded the minimum mean lesion size of 2.23 cm^2^ while ST recorded a maximum of 4.16 cm^2^ whereas in between the endophytes, treatment with strain A6 and PGSS1 recorded a minimum mean lesion size of 2.40 cm^2^ and 2.41 cm^2^ respectively (exhibiting decrease of 2.37-fold over negative control (5.69 cm^2^)) whereas HP3d recorded a maximum of 2.76 cm^2^ (showing a decrease by 2.06- fold compared to negative control). Compared to endophytes treatment, *P*. *fluorescence* (positive control) recorded a maximum mean lesion size of 2.91 cm^2^ (Supplementary Table 6; Fig. 1B). All strains recorded minimum lesion size compared to negative control, thus showing their biocontrol efficacy in reducing lesion formation.

The severity of sheath blight disease was assessed for each treatment individually by dividing the lesion height by plant height and expressed in percentage [15]. ST + SD + FS recorded a minimum mean PDI of 3.64, whereas ST recorded a maximum of 6.79. Treatment with strain A6 and PGSS1 recorded a minimum mean PDI of 3.82 and 3.86 respectively thus exhibiting a decrease in sheath blight intensity by 2.60 and 2.58fold over negative control (9.95 PDI), whereas with HP3d recorded a maximum of 4.38 and 2.27-fold decrease over negative control. *P. fluorescence* was less effective than the tested endophytes with a maximum PDI of 4.68. Strains P42, HP3d, PGSS1, A6 and *P. fluorescence* in ST + SD + FS treatment recorded minimum mean PDI of 2.28, 2.59, 1.86, 1.92 and 3.37 respectively over control (9.95) (Supplementary Table 7; Fig. 1B).

### 3.3 Enzyme analysis and chlorophyll estimation

The administration of the four endophytic strains A6, P42, PGSS1 and HP3d to rice was implemented through methods *viz*., ST @10 mL/kg, SD @10 mL/L of water, FS @10 mL/L of water, and their combinations resulted in varied PPO, POD, SOD, and PAL activity against the sheath blight pathogen. The analysis done evinced that the activity of all the enzymes was elicitated significantly in all 30 treatments compared to the uninoculated control. Furthermore, enzymes gradually increased until the fourth day after challenged inoculation and then declined.

#### 3.3.1 Polyphenol oxidase (PPO) activity

**Fig. 2:**
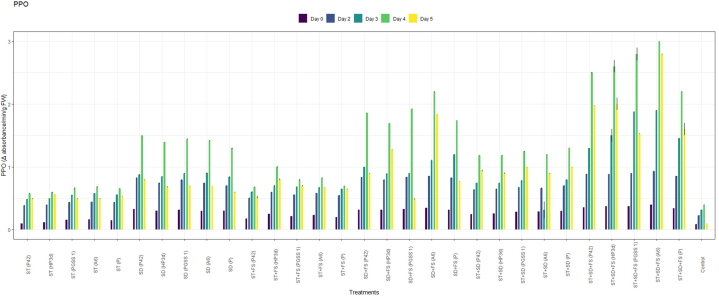

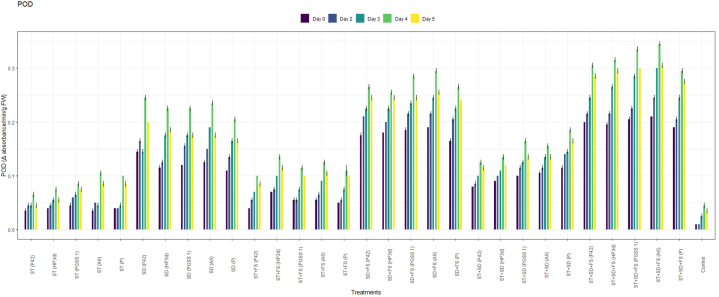

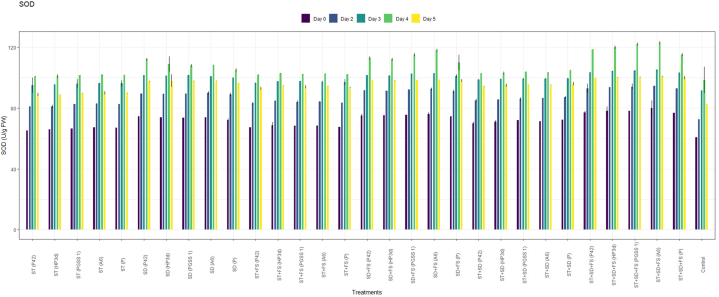

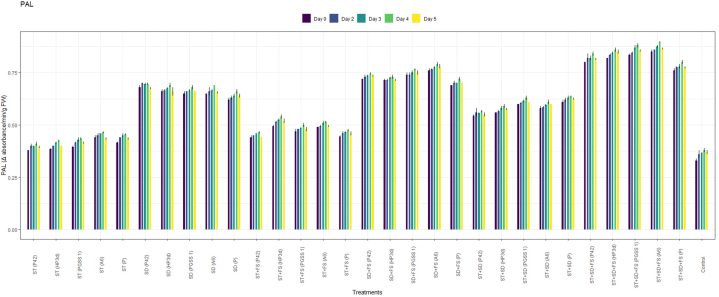
Effect of different treatments of bacterial endophytes on (a) PPO (Change in absorbance min^−1^ g ^−1^), (b) POD (Change in absorbance min^−1^g ^−1^ FW), (c) SOD (Ug^−1^FW), and (d) PAL (Change in absorbance min^−1^g ^−1^FW), activity on challenged inoculation with *R. solani* in rice plants under glasshouse conditions. The figures presented are the mean values of three replications. Standard errors of the mean values are presented as bars.

PPO analysis revealed a significant increased activity across 30 treatments when compared to the uninoculated control as shown in Fig. 2a. Among the treatments, the highest activity of PPO was recorded in the treatment, ST + SD + FS with strain A6 (3.00 U^−1^min^−1^g^−1^) followed by strain PGSS1 (2.80 U^−1^min^−1^g^−1^), HP3d (2.60 U^−1^min^−1^g^−1^), P42 (2.50 U^−1^min^−1^g^−1^) and *P*. *fluorescence* (2.20 U^−1^min^−1^g^−1^) over uninoculated control (0.4 U^−1^min^−1^g^−1^) on 4th day after challenged inoculation with *R. solani* thus exhibiting an increase of 7.5, 7, 6.5, 6.25 and 5.5 fold, respectively over uninoculated control.

#### 3.3.2 Peroxidase (POD) activity

For POD, all 30 treatments recorded a significant increase in activity compared to uninoculated control as shown in Fig. 2b and the highest elicitation was shown in the treatment, ST + SD + FS with strains A6 (0.35 min^−1^g^−1^) and PGSS1 (0.34 min^−1^g^−1^) exhibiting significant increase of 7- and 6.8- fold respectively over uninoculated control (0.05 U^−1^min^−1^g^−1^) but were on par with positive control, *P*. *fluorescence* (0.30 min^−1^g^−1^) on 4th day after challenged inoculation with *R. solani*.

#### 3.3.3 Superoxidase dismutase (SOD) activity

In the case of SOD, all 30 treatments showed an increase in activity compared to the control on all days (Fig. 2c**)**, among which the treatment involving ST + SD + FS with strain A6 exhibited the highest activity with a measured value of 123 Ug^−1^ FW. Similarly, PGSS1, HP3d, and P42 demonstrated a substantial on-par increase in activity with A6 i.e., 122 Ug^−1^ FW, 120 Ug^−1^ FW and 118 Ug^−1^ FW respectively. These values represented a significant increase over the uninoculated control, showing 1.25, 1.24, 1.22 and 1.20 fold increases respectively. Moreover, these results were statistically significantly higher compared to positive control *P. fluorescence* (115 Ug-1 FW) on the 4th day following the challenged inoculation *with R. solani*.

#### 3.3.4 Phenylalanine ammonia lyase (PAL) activity

The treatment involving ST + SD + FS with strains A6 (0.90 min⁻^1^g⁻^1^FW) and PGSS1 (0.88 min⁻^1^g⁻^1^FW) demonstrated the highest PAL activity among all the 30 treatments, both exhibiting 2.37- and 2.32-fold increases compared to the uninoculated control (0.38 Ug⁻^1^ FW). Moreover, these values substantially increased compared to the PAL activity observed in *P. fluorescence* (0.80 min⁻^1^g⁻^1^FW) on the 4th day after being inoculated with *R. solani* (Fig. 2d).

### 3.4 Chlorophyll estimation

**Fig. 3:**
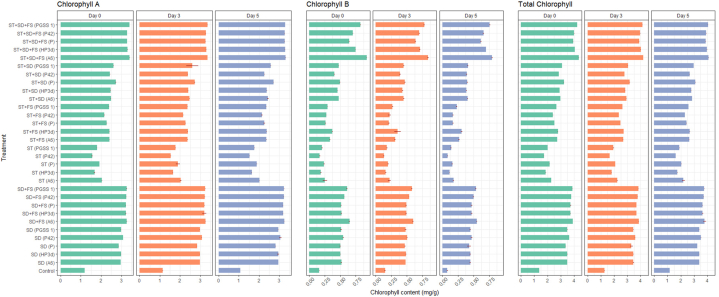
Effect of different treatments of bacterial endophytes on chlorophyll content (mg/g) on 0th, 3rd and 5th day after challenged inoculation with *R. solani* in rice plants under glasshouse conditions. The figures presented are the mean values of three independent experiments. Standard errors of the mean values are presented as bars.

Chlorophyll content was estimated in rice on the 0^th^, 3rd and 5th day after challenge inoculation with *R. solani*. Among 30 treatments and at various time intervals (days), endophytes inoculated sheath blight infected plants recorded significantly higher chlorophyll *a*, chlorophyll *b*, and total chlorophyll concentration compared to uninoculated control. The concentration was higher on the 0^th^ day, dropped on the 3rd day, and was lowest on the 5th day as shown in Fig. 3*.* The highest content of chlorophyll *a*, chlorophyll *b* and total chlorophyll was exhibited in the treatment, ST + SD + FS with endophytes, among which plants subjected to *B. velezensis* strain A6 showed the highest chlorophyll *a*, chlorophyll *b* and total chlorophyll of 3.41 mg/g, 0.93 mg/g and 4.35 mg/g respectively which was significant over *P. fluorescens* bearing 3.28 mg/g,0.65 mg/g and 3.93 mg/g and uninoculated control having 1.19 mg/g, 0.17 mg/g and 1.38 mg/g respectively on 0^th^ day after challenge inoculation with *R. solani*. Thus ST + SD + FS with *B. velezensis* strain A6 exihibited 2.87, 5.47 and 3.15 folds less decrease in chlorophyll *a*, chlorophyll *b* and total chlorophyll over uninoculated control. The lowest concentration was recorded in ST with endophytes (Fig. 3).

### 3.5 Field evaluation of bacterial endophytes against sheath blight of rice

Based on positive results in *in vitro* experiments *i.e.*, endophytes effectiveness in promoting plant growth and increase in antioxidant enzymes activity upon challenge inoculation with sheath blight pathogen; field studies were undertaken to check their efficacy *in vivo* conditions during the summer of 2021. Each of the four endophytes, positive control (*P. fluorescence*) and recommended fungicide (Hexaconazole 5SC) were tested in different combinations. Among the evaluated 22 treatments, Hexaconazole 5SC showed the significantly highest average shoot growth, measuring 83.33 cm. In contrast, the control treatment exhibited the lowest shoot growth of 67.00 cm. The remaining treatments with endophyte inoculation demonstrated similar average shoot growth, with no significant differences. However, they consistently displayed significantly greater shoot length in comparison to the uninoculated control *i.e.*, treatment ST + SD + FS with bacterial endophytes A6, P42, PGSS1 and HP3d showed 1.2, 1.1,1.2 and 1.1 fold increase in shoot length over uninoculated control. The treatment ST + SD + FS (P42) recorded the highest, exhibiting 1.39-fold increase in the mean number of tillers. No statistically significant differences were observed among the remaining treatments, compared to each other and in relation to the control treatment.

**Fig. 4:**
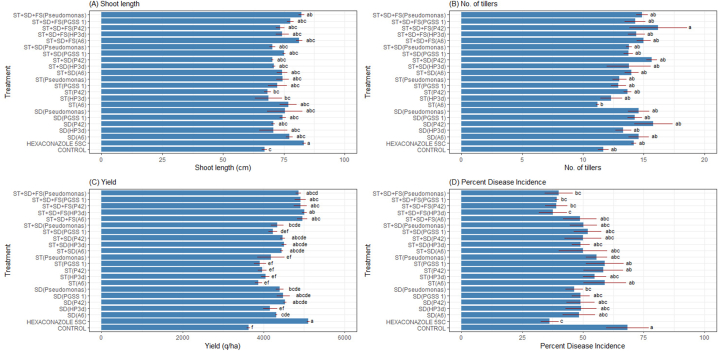
Effect of different endophytes treatments on the (A) shoot length (cm), (B) no. of tillers, (C) yield (kg/ha) and (D) sheath blight intensity of rice under field conditions. The figures presented are the mean values of three independent experiments. Standard errors of the mean values are presented as bars. Letters on bars indicate DMRT at *p* < 0.05.

All treatments exhibited statistically significant differences compared to the uninoculated control in PDI. The lowest PDI and the highest PDOC were observed in treatments Hexaconazole 5SC, ST + SD + FS(HP3d), ST + SD + FS(Pseudomonas), ST + SD + FS(P42), and ST + SD + FS (PGSS 1) exhibiting 1.88-, 1.81-, 1.71-, 1.75- and 1.74- fold decrease in PDI compared to uninoculated control. In contrast, the highest PDI and the lowest PDOC were observed in treatments ST(P42), ST (PGSS 1), and ST(A6). Variations in yield were apparent among the treatments, with Hexaconazole 5SC showing the highest average yield of 5098.89 kg/ha. Among endophytes, treatments including ST + SD + FS(HP3d), ST + SD + FS (PGSS1), ST + SD + FS(P42), and ST + SD + FS(A6) formed a distinct group with relatively high yields exhibiting 1.38, 1.35, 1.35 and 1.36 fold increase in yield over uninoculated control. On the other hand, treatments like SD (PGSS 1), ST + SD (PGSS 1), and ST + SD (*P. fluorescence*) displayed lower yields, and the control treatment had the lowest mean yield of 3626.67 kg/ha. The remaining treatments showcased insignificantly similar yields (Supplementary Table 8; Fig. 4).

## 4 Discussion

Our previous studies with various molecular methods and biochemical tests identified *B. velezensis* strains A6 and P42 as potential biocontrol agents [12,13]. The present study focused on the characterized *B. velezensis* strains P42 and A6 and novel BCAs *B. pseudomycoides* strain HP3d and *P. polymyxa* strain PGSS1, involvement in PGPR activity, their antagonistic potential and ISR-related antioxidant enzymes *viz.*, PPO, POD, SOD and PAL activities against sheath blight in rice both *in vivo* and *in vitro* conditions.

PGP attributes *viz.*, ammonia production, siderophore production, IAA production, and phosphorus solubilization were assessed for the four endophytes *in vitro* conditions. Ammonia production and phosphorus solubilization represent key traits among endophytes that facilitate the growth of associated plants. All tested endophytes were shown to be positive for both traits and showed the range of production. Siderophores are compounds released by microbes in response to limited iron availability in the soil and thus can access and utilize soluble Fe^3+^-siderophore complexes for their iron requirements [33]. In the present study, all four endophytes produced siderophores, as evidenced by the formation of yellow zones on the CAS agar medium plate and thus may be modulating plant growth by either direct supply of iron to plants or indirectly by depriving fungal pathogens of iron [34]. While plants are capable of synthesizing their phytohormones, they can also benefit from external sources produced by plant-associated bacteria and fungi, such as *Bacillus* spp. In the present study, all four endophytes were able to produce substantial amounts of IAA, ranging from 15.20 to 22.19 μg mL^−1^ which typically serve as a plant growth promoter by producing auxin [33]. These findings align with previous studies of [16,35], who obtained similar findings.

The effect of bacterial endophytes on PGP activities and ISR in rice after challenge inoculation with *R. solani* was investigated under glasshouse conditions. When compared to the uninoculated control, all endophytes inoculated plants had considerably longer shoots, roots, number of tillers, and percent root and shoot dry weight, due to triggered production of phytohormones like auxins, gibberellins, and cytokinins [12]. In addition, compared to uninoculated control, the tested endophytes were beneficial in reducing lesion size and sheath blight disease intensity. The results were in line with the outcome of studies performed by Refs. [9,26] who noted the influence of endophytes on PGP and ISR in rice against sheath blight. For each of the four bacterial endophytes, the most effective combination of treatments was recorded to be ST + SD + FS, followed by SD + FS and SD, and ST was shown to be the least effective. This indicates that endophytes used to control sheath blight infection need to colonize the paddy stem surface above the waterline, suggesting that bioagents applied as an SD and combined ST + SD + FS will be highly effective against *R. solani* infection as the initial establishment and robustness of the bioagent in the rhizosphere/rhizoplane and phylloplane will influence the microclimate to its advantage, dominating the environment, thus inducing disease resistance in plants.

In general defense genes against phytopathogens are present in plants in quiescent and get activate and induce systemic resistance by activation of various defense-related enzymes *viz.*, PO, PPO, PAL, chitinase and β −1,3-glucanase, under various biotic constraints [14,36]. In present study, the PPO, POD, SOD, and PAL enzyme activity were measured in rice after treatments with different endophytes *via* different modes, following challenge inoculation with *R. solani* where ST + SD + FS was the optimal treatment, followed by SD + FS and SD, with ST being the least effective. PPO is a crucial metalloproteinase that catalyzes the oxidation of phenols to quinones, synthesizing melanin and cross-linked protein polymers. It plays a vital role in defending against biotic and abiotic stresses and is significant in repairing membrane damage caused by stress and in strengthening cell membranes [37]. POD are recognized for their dual role in hardening or softening plant cell walls in plant growth. They are involved in various processes, including germination, lignification, development, and plant defense. Their mechanisms of action include radical formation, ROS regulation, and substrate oxidation [38]. SOD serves as the main defense mechanism against reactive oxygen species (ROS). SOD iso-enzymes play critical roles in protecting cells from the toxic effects of superoxide radicals produced at various cellular loci [39]. PAL is an essential enzyme that catalyzes the deamination of phenylalanine to *trans*-cinnamate. This reaction is part of a secondary metabolite synthesis pathway that produces phenolic compounds, including anthocyanins, hormones, phytoalexins, flavonoids, isoflavonoids and lignin which are crucial for plant defense against biotic stresses [40]. The combined production of these biologically active compounds by the endophytes likely contributes to the increased bioefficacy of the cultivar against *R. solani*. Also, on the 0^th^, 2nd, 3rd, 4th, and 5th day following challenge inoculation with *R. solani*, we found the activity of these enzymes to gradually increase up to the fourth day and then decline. Thus, it was noted that the timing and expression patterns of these defense mechanisms were crucial for effectively suppressing the pathogen [14,41]. The increased functioning of these antioxidant enzymes can be attributed to their role as agents that counteract reactive oxygen species (which serve to mitigate the effect of pathogen invasion) by detoxifying these harmful molecules [42,43]. Chlorophyll content measurements were also conducted after challenge inoculation with *R. solani*, on the 0^th^, 3rd and 5th day and were found to show considerably lower chlorophyll *a*, chlorophyll *b*, and total chlorophyll content, which further declined with time. However, the reduction in plants subjected to endophytes was lower than the uninoculated control. The treatment ST + SD + FS had the highest content of chlorophyll *a*, chlorophyll *b*, and total chlorophyll, while the treatment ST had the lowest. The reduction in chlorophyll *a*, chlorophyll *b*, and total chlorophyll could be attributed to chlorophyll degradation associated with the leaf blight symptoms [44]. The findings align with that of [45], who recorded higher chlorophyll content as compared to positive and negative control on application of *Bacillus subtilis* (BS-01, BS-02) and *Pseudomonas maltophilia* (PM-01).

In the field studies, all plots with endophytic treatments showed enhanced shoot length, number of tillers, yield, and low PDI against sheath blight than the uninoculated control, and were comparable to the hexaconazole. Among treatments ST @10 mL/kg + SD@10 mL/L + FS @10 mL/L of water showed the best results. Hence, when used as fresh suspensions, the endophytes have the potential to promote plant growth and manage sheath blight disease. Similarly, several scientists have reported the management of phytopathogens by different bacterial endophytes. Prasanna Kumar et al. (2017), reported reduced intensity of diseases *viz.* blast, sheath blight and bacterial blight and increased grain yield in a paddy on treatment with *B. subtilis* strains [46]. Similarly, Chen et al. (2020) [47] tested the antagonistic activity of *B. velezensis* strain ZW-10 against *Magnaporthe oryzae* and recorded a significant inhibitory effect on rice blast in field conditions. However, several factors may have an influence on the biological activity of endophytic bacteria under field conditions including the competence of bacteria to colonize the plant, the host plant's characteristics and environmental conditions. Key determinants include the plant's age, genotype, geographical location, growth stage, and specific tissues analyzed. Climatic conditions also play a significant role, in affecting the abundance and composition of endophytic bacteria. Soil type influences endophytic diversity, as the same plant cultivar can harbor different endophytes when grown in varied soils. Additionally, plants can selectively recruit endophytic bacteria based on stress factors such as the presence of phytopathogens. This selection process is dynamic and closely controlled by the host plant to favor bacteria beneficial to its growth and defense [48].

A multitude of work has been done to assess the efficacy of endophytes *in vitro* however, because of above mentioned reasons many of these studies do not translate to successful results in field conditions. Ours is one of the studies that demonstrated optimal results for all the diverse crop-derived endophytes in the laboratory, glasshouse and field conditions. As the effectiveness of our endophytes is evident *in vitro* and *in vivo* studies, through a specialized model system for complex multipartite interactions and by recognizing and categorizing the plenitude of factors in plants and environment which generally reduce endophyte efficacy, their antagonistic potential can be efficiently utilized in future for the suppression of the sheath blight pathogen.

## 5 Conclusion

This study has shown that our strains P42, A6, HP3d and PGSS1 isolated from diverse crops and evaluated through different methods of applications both *in vivo* and *in planta* conditions, showed their non-host specific nature thus exhibiting broad-spectrum activity. The endophytes induced PGP and ISR activity against sheath blight pathogen in rice plants by modulation of various defense-related enzymes *viz.*, PO, PPO, PAL, and SOD which eventually resulted in higher yield. Among the treatments, ST + SD + FS was the most optimum treatment that can be employed in the field to raise the quality of rice. Therefore, we conclude that our strains can prove effective in ameliorating the productivity of rice crop and as endophytes usually do not have biocidal activity and are environmentally sound with a lower risk of pathogen resistance development, it is expected that results reported in this study will lead to an understanding of the effective role of non-host endophytes in managing diseases in a wide range of crops. Future research including underlying molecular mechanisms driving the observed PGPR and ISR activity, interaction between introduced endophytes and native microbial communities and application methods for large-scale field use, including formulation and delivery could pave the way for utilizing these endophytes as efficient, cost-effective and environmentally friendly bioformulations in the rice ecosystem.

## Ethical statement

No human or animal participants were involved in this study.

## Funding

For the publication of this manuscript, funding was provided by Researchers Supporting Project number (RSP2024R96).

## Consent for publication

All authors have approved for publication.

## Data availability statement

The data used to support the findings of this study were included in the article.

## CRediT authorship contribution statement

**Aditya Kukreti:** Writing – original draft, Visualization, Validation, Software, Methodology, Investigation, Formal analysis, Data curation. **Chethana Bangi Siddabasappa:** Writing – review & editing, Supervision, Resources, Investigation, Conceptualization. **Prasannakumar Muthakapalli Krishnareddy:** Writing – review & editing, Supervision, Project administration, Investigation, Conceptualization. **Yashavanth Basavapatna Subbanna:** Visualization, Software. **Manjunatha Channappa:** Writing – review & editing, Validation, Supervision. **Shivakumara Kadanakuppe Thammayya:** Writing – review & editing, Validation, Supervision. **Eman A. Mahmoud:** Writing – review & editing, Funding acquisition. **Rafa Almeer:** Writing – review & editing, Funding acquisition.

## Declaration of competing interest

The authors declare that they have no known competing financial interests or personal relationships that could have appeared to influence the work reported in this paper.
