# Supplementary material for: Impact of the tripartite interaction between rice, sheath blight and diverse crop-associated endophytes on phenotypic and biochemical responses in rice
Source: Heliyon. 2024 Jun 6;10(12):e32574. doi: 10.1016/j.heliyon.2024.e32574 (PMC11341311; doi:10.1016/j.heliyon.2024.e32574)
Supplement: Multimedia component 1 [file mmc1.docx]

**Supplementary table 1:** Effect of bacterial endophytes on shoot length (cm) of rice under glasshouse conditions by two-way ANOVA

| **TREATMENTS** | **ENDOPHYTES** | | | | |  |  |
| --- | --- | --- | --- | --- | --- | --- | --- |
|  | **P42** | **HP3d** | **PGSS-1** | **A6** | **Pseudomonas**  **(positive control)** | **Negative control** | **Mean** |
| **Seed treatment** | 58.78 | 56 | 57.11 | 60.78 | 55.22 | 57.56 | 57.58 |
| **Seedling dip** | 65.11 | 64.23 | 65.00 | 63.22 | 63.33 | 57.67 | 63.09 |
| **Seed treatment +foliar spray** | 59.22 | 63.22 | 60.67 | 62.78 | 60.56 | 58.00 | 60.80 |
| **Seedling dip+ foliar spray** | 65.78 | 65.34 | 65.11 | 65.11 | 66.44 | 57.23 | 64.09 |
| **Seed treatment + seedling dip** | 64.78 | 63.56 | 61.11 | 63 | 62.99 | 57.00 | 61.63 |
| **Seed treatment + seedling dip+ foliar spray** | 66.00 | 66.00 | 66.67 | 65.78 | 66.45 | 57.89 | 64.93 |
| **Mean** | 63.28 | 63.06 | 62.61 | 63.45 | 62.50 | 57.22 |  |
|  | **CD at 1%** | **CD at 5%** |  |  |  |  |  |
| **Treatments (T)** | 0.89 | 0.67 |  |  |  |  |  |
| **Endophytes (E)** | 0.89 | 0.67 |  |  |  |  |  |
| **TXE** | 2.19 | 1.65 |  |  |  |  |  |

**Supplementary table 2:** Effect of bacterial endophytes on root length (cm) of rice under glasshouse conditions by two-way ANOVA

| **TREATMENTS** | **ENDOPHYTES** | | | | |  |  |
| --- | --- | --- | --- | --- | --- | --- | --- |
|  | **P42** | **HP3d** | **PGSS-1** | **A6** | **Pseudomonas**  **(positive control)** | **Negative control** | **Mean** |
| **Seed treatment** | 14.23 | 11.00 | 11.00 | 13.00 | 13.67 | 14.17 | 12.85 |
| **Seedling dip** | 17.17 | 15.33 | 18.17 | 16.83 | 18.33 | 15.00 | 16.81 |
| **Seed treatment +foliar spray** | 14.33 | 13.33 | 13.83 | 16.00 | 18.50 | 15.00 | 15.17 |
| **Seedling dip+ foliar spray** | 17.83 | 15.83 | 20.00 | 21.17 | 20.00 | 15.17 | 18.33 |
| **Seed treatment + seedling dip** | 17.67 | 14.67 | 17.00 | 16.67 | 19.00 | 14.83 | 16.64 |
| **Seed treatment + seedling dip+ foliar spray** | 23.77 | 21.00 | 26.67 | 21.33 | 22.33 | 14.83 | 21.66 |
| **Mean** | 17.50 | 15.19 | 17.78 | 17.50 | 18.64 | 14.83 |  |
|  | **CD at 1%** | **CD at 5%** |  |  |  |  |  |
| **Treatments (T)** | 0.51 | 0.39 |  |  |  |  |  |
| **Endophytes (E)** | 0.51 | 0.39 |  |  |  |  |  |
| **TXE** | 1.26 | 0.95 |  |  |  |  |  |

| **TREATMENTS** | **ENDOPHYTES** | | | | |  |  |
| --- | --- | --- | --- | --- | --- | --- | --- |
|  | **P42** | **HP3d** | **PGSS-1** | **A6** | **Pseudomonas**  **(positive control)** | **Negative control** | **Mean** |
| **Seed treatment** | 3.22 | 2.78 | 2.78 | 3.33 | 2.89 | 2.86 | 2.98 |
| **Seedling dip** | 3.89 | 3.89 | 5.00 | 4.56 | 4.15 | 2.87 | 4.06 |
| **Seed treatment +foliar spray** | 3.56 | 3.22 | 3.89 | 4.11 | 3.67 | 2.89 | 3.56 |
| **Seedling dip+ foliar spray** | 4.00 | 4.17 | 5.44 | 5.45 | 4.78 | 2.85 | 4.45 |
| **Seed treatment + seedling dip** | 3.67 | 3.33 | 4.00 | 4.45 | 3.97 | 2.86 | 3.71 |
| **Seed treatment + seedling dip+ foliar spray** | 6.22 | 4.34 | 6.22 | 5.55 | 6.33 | 2.90 | 5.26 |
| **Mean** | 4.09 | 3.62 | 4.56 | 4.58 | 4.30 | 2.87 |  |
|  | **CD at 1%** | **CD at 5%** |  |  |  |  |  |
| **Treatments (T)** | 0.17 | 0.13 |  |  |  |  |  |
| **Endophytes (E)** | 0.17 | 0.13 |  |  |  |  |  |
| **TXE** | 0.42 | 0.31 |  |  |  |  |  |

**Supplementary table 3:** Effect of endophytes treatments on rice no. of tillers per plant under glasshouse conditions by two-way ANOVA

**Supplementary table 4:** Effect of bacterial endophytes on % shoot dry matter of rice under glasshouse conditions by two-way ANOVA

| **TREATMENTS** | **ENDOPHYTES** | | | | |  |  |
| --- | --- | --- | --- | --- | --- | --- | --- |
|  | **P42** | **HP3d** | **PGSS-1** | **A6** | **Pseudomonas**  **(positive control)** | **Negative control** | **Mean** |
| **Seed treatment** | 76.00  (60.67) | 77.73  (61.57) | 78.68  (62.47) | 77.55  (61.72) | 70.00  (57.00) | 80.34  (63.68) | 76.64  (61.19) |
| **Seedling dip** | 80.78  (64.00) | 80.98  (64.15) | 82.55  (65.31) | 82.80  (65.50) | 82.67  (65.40) | 79.40  (63.01) | 81.53  (64.56) |
| **Seed treatment +foliar spray** | 78.36  (62.28) | 78.34  (62.26) | 81.45  (64.69) | 81.99  (64.89) | 76.62  (61.08) | 79.93  (63.38) | 79.45  (63.10) |
| **Seedling dip+ foliar spray** | 83.72  (66.20) | 81.24  (64.33) | 82.70  (65.42) | 84.37  (66.71) | 83.67  (66.17) | 79.00  (62.73) | 82.45  (65.26) |
| **Seed treatment + seedling dip** | 79.46  (63.05) | 80.90  (64.08) | 81.92  (64.84) | 82.67  (65.40) | 78.62  (62.46) | 80.33  (63.67) | 80.65  (63.92) |
| **Seed treatment + seedling dip+ foliar spray** | 87.36  (69.17) | 84.66  (66.94) | 84.86  (67.10) | 86.43  (68.38) | 88.83  (70.48) | 79.37  (63.80) | 85.25  (67.15) |
| **Mean** | 80.95  (64.23) | 80.58  (63.89) | 82.02  (64.97) | 82.64  (65.43) | 80.07  (63.77) | 79.73  (62.88) |  |
|  | **CD at 1%** | **CD at 5%** |  |  |  |  |  |
| **Treatments (T)** | 1.84 | 1.38 |  |  |  |  |  |
| **Endophytes (E)** | 1.84 | 1.38 |  |  |  |  |  |
| **TxE** | 4.50 | 3.39 |  |  |  |  |  |

*Values in parentheses are arcsine-transformed values

**Supplementary table 5:** Effect of bacterial endophytes on % root dry matter of rice under glasshouse conditions by two-way ANOVA

| **TREATMENTS** | **ENDOPHYTES** | | | | |  |  |
| --- | --- | --- | --- | --- | --- | --- | --- |
|  | **P42** | **HP3d** | **PGSS-1** | **A6** | **Pseudomonas**  **(positive control)** | **Negative control** | **Mean** |
| **Seed treatment** | 57.26  (49.17) | 51.71  (45.98) | 51.33  (45.76) | 52.38  (46.36) | 52.56  (46.47) | 54.90  (47.81) | 53.36  (46.93) |
| **Seedling dip** | 68.16  (55.65) | 65.34  (53.93) | 68.33  (55.75) | 66.33  (54.33) | 66.20  (54.45) | 54.00  (47.30) | 64.73  (53.57) |
| **Seed treatment +foliar spray** | 66.33  (54.53) | 52.41  (46.38) | 55.69  (48.27) | 55.78  (48.32) | 54.33  (47.48) | 54.67  (47.68) | 56.54  (48.78) |
| **Seedling dip+ foliar spray** | 70.00  (56.80) | 67.19  (55.05) | 77.48  (61.67) | 68.57  (55.90) | 68.28  (55.72) | 55.00  (47.87) | 67.75  (55.50) |
| **Seed treatment + seedling dip** | 66.80  (54.82) | 57.12  (49.09) | 57.00  (49.02) | 59.02  (50.20) | 58.96  (50.16) | 55.00  (47.87) | 58.98  (50.19) |
| **Seed treatment + seedling dip+ foliar spray** | 71.44  (57.70) | 68.28  (55.74) | 79.17  (62.85) | 69.97  (56.77) | 70.12  (56.86) | 55.00  (47.87) | 69.00  (56.30) |
| **Mean** | 66.66  (54.78) | 60.34  (51.03) | 64.83  (53.89) | 62.01  (51.98) | 61.74  (51.86) | 54.67  (47.73) |  |
|  | **CD at 1%** | **CD at 5%** |  |  |  |  |  |
| **Treatments (T)** | 0.42 | 0.32 |  |  |  |  |  |
| **Endophytes (E)** | 0.42 | 0.32 |  |  |  |  |  |
| **TXE** | 1.03 | 0.78 |  |  |  |  |  |

*Values in parentheses are arcsine-transformed values

**Supplementary table 6:** Effect of endophytes treatments on size of lesion (l x w cm^2^) under glasshouse conditions by two-way ANOVA

| **TREATMENTS** | **ENDOPHYTES** | | | | |  |  |
| --- | --- | --- | --- | --- | --- | --- | --- |
|  | **P42** | **HP3d** | **PGSS-1** | **A6** | **Pseudomonas**  **(positive control)** | **Negative control** | **Mean** |
| **Seed treatment** | 4.09 | 4.04 | 3.91 | 3.39 | 3.85 | 5.65 | 4.16 |
| **Seedling dip** | 2.01 | 2.81 | 1.93 | 2.12 | 2.64 | 5.67 | 2.86 |
| **Seed treatment +foliar spray** | 3.93 | 3.04 | 3.27 | 3.07 | 3.57 | 5.90 | 3.76 |
| **Seedling dip+ foliar spray** | 1.75 | 2.13 | 1.64 | 1.83 | 2.46 | 5.68 | 2.58 |
| **Seed treatment + seedling dip** | 3.00 | 2.82 | 2.51 | 2.76 | 2.81 | 5.50 | 3.27 |
| **Seed treatment + seedling dip+ foliar spray** | 1.35 | 1.71 | 1.19 | 1.25 | 2.14 | 5.77 | 2.23 |
| **Mean** | 2.69 | 2.76 | 2.41 | 2.40 | 2.91 | 5.69 |  |
|  | **CD at 1%** | **CD at 5%** |  |  |  |  |  |
| **Treatments (T)** | 0.13 | 0.10 |  |  |  |  |  |
| **Endophytes (E)** | 0.13 | 0.10 |  |  |  |  |  |
| **TXE** | 0.31 | 0.24 |  |  |  |  |  |

**Supplementary table 7:** Effect of endophytes treatments on sheath blight severity (%) under glasshouse conditions by two-way ANOVA

| **TREATMENTS** | **ENDOPHYTES** | | | | |  |  |
| --- | --- | --- | --- | --- | --- | --- | --- |
|  | **P42** | **HP3d** | **PGSS-1** | **A6** | **Pseudomonas**  **(positive control)** | **Negative control** | **Mean** |
| **Seed treatment** | 6.29  (14.53) | 6.97  (15.30) | 6.31  (14.55) | 5.57  (13.66) | 5.75  (13.88) | 9.82  (18.26) | 6.79  (15.03) |
| **Seedling dip** | 3.03  (10.03) | 4.26  (11.92) | 3.26  (10.40) | 3.38  (10.60) | 3.97  (11.49) | 9.83  (18.27) | 4.62  (12.12) |
| **Seed treatment +foliar spray** | 5.67  (13.77) | 4.64  (12.44) | 4.90  (12.79) | 4.86  (12.73) | 5.90  (14.06) | 10.11  (18.54) | 5.95  (13.99) |
| **Seedling dip+ foliar spray** | 3.13  (10.19) | 3.35  (10.54) | 2.70  (9.45) | 2.79  (9.61) | 4.00  (11.54) | 10.01  (18.44) | 4.33  (11.63) |
| **Seed treatment + seedling dip** | 4.64  (12.43) | 4.46  (12.19) | 4.11  (11.69) | 4.39  (12.09) | 5.08  (13.03) | 10.12  (18.55) | 5.53  (13.39) |
| **Seed treatment + seedling dip+ foliar spray** | 2.28  (8.86) | 2.59  (9.25) | 1.86  (7.83) | 1.92  (7.95) | 3.37  (10.58) | 9.83  (18.28) | 3.64  (10.46) |
| **Mean** | 4.17  (11.61) | 4.38  (11.94) | 3.86  (11.12) | 3.82  (11.11) | 4.68  (12.43) | 9.95  (18.39) |  |
|  | **CD at 1%** | **CD at 5%** |  |  |  |  |  |
| **Treatments (T)** | 0.31 | 0.24 |  |  |  |  |  |
| **Endophytes (E)** | 0.31 | 0.24 |  |  |  |  |  |
| **TXE** | 0.76 | 0.58 |  |  |  |  |  |

*Values in parentheses are arcsine-transformed values

**Supplementary table 8:** Effect of different endophytes treatments on the plant growth parameters and sheath blight intensity of rice under field conditions

|  | **GROWTH PARAMETERS** | | **DISEASE INTENSITY** | |  |
| --- | --- | --- | --- | --- | --- |
| **TREATMENTS** | **Shoot**  **length 80 DAT**  **(cm)** | **No. of tillers**  **80 DAT** | **PDI*** | **PDOC** | **Yield****  **(kg/ha)** |
| \| **ST(P42)** \| \| --- \| | 68.27^bc^ | 13.67^ab^ | 58.41 (49.94) ^ab^ | 14.61 | 3966.11(62.97) ^ef^ |
| \| **SD(P42)** \| \| --- \| | 70.60^abc^ | 15.80^ab^ | 49.00 (44.43) ^bcde^ | 28.36 | 4525.00(67.27) ^abcde^ |
| \| **ST+SD(P42)** \| \| --- \| | 70.27^abc^ | 15.67^ab^ | 50.00 (44.97) ^bcd^ | 26.90 | 4467.78(66.85) ^abcde^ |
| \| **ST+SD+FS(P42)** \| \| --- \| | 73.51^abc^ | 16.20^a^ | 39.07 (38.62) ^cde^ | 42.87 | 4906.67(70.03) ^abc^ |
| \| **ST(HP3d)** \| \| --- \| | 68.67^bc^ | 12.33^ab^ | 54.73 (47.73) ^b^ | 19.99 | 4043.89(63.59) ^ef^ |
| \| **SD(HP3d)** \| \| --- \| | 70.67^abc^ | 13.33^ab^ | 49.26 (44.56) ^bcde^ | 27.98 | 4161.67(64.49) ^efgh^ |
| \| **ST+SD(HP3d)** \| \| --- \| | 70.93^abc^ | 13.77^ab^ | 49.07 (44.47) ^bcde^ | 28.25 | 4500.33(67.08) ^abcde^ |
| \| **ST+SD+FS(HP3d)** \| \| --- \| | 74.33^abc^ | 14.40^ab^ | 37.67 (37.77) ^de^ | 44.93 | 5000.56(70.72) ^ab^ |
| **ST(PGSS1)** | 72.33^abc^ | 12.93^ab^ | 59.01(50.32)^ab^ | 13.72 | 3909.33(62.51) ^ef^ |
| \| **SD (PGSS 1)** \| \| --- \| | 74.53^abc^ | 14.27^ab^ | 49.07(44.47) ^bcde^ | 28.25 | 4483.33(66.94) ^abcde^ |
| \| **ST+SD (PGSS 1)** \| \| --- \| | 75.00^abc^ | 13.73^ab^ | 51.91(46.10) ^bc^ | 24.10 | 4230.00(65.04) ^def^ |
| \| **ST+SD+FS (PGSS 1)** \| \| --- \| | 77.67^abc^ | 14.30^ab^ | 39.26(38.80) ^cde^ | 42.60 | 4905.56(70.03) ^abc^ |
| \| **ST(A6)** \| \| --- \| | 76.80^abc^ | 11.20^b^ | 59.01(50.33) ^ab^ | 13.72 | 3872.00(62.22) ^ef^ |
| \| **SD(A6)** \| \| --- \| | 77.20^abc^ | 14.60^ab^ | 48.33(44.00 ^bcde^ | 29.34 | 4313.33(65.68) ^cde^ |
| \| **ST+SD(A6)** \| \| --- \| | 74.27^abc^ | 14.00^ab^ | 50.00(44.92) ^bcd^ | 26.90 | 4442.67(66.66) ^abcde^ |
| \| **ST+SD+FS(A6)** \| \| --- \| | 81.33^abc^ | 15.00^ab^ | 48.77(44.27) ^bcde^ | 28.71 | 4954.44(70.38) ^abc^ |
| \| **ST (PF)** \| \| --- \| | 74.47^abc^ | 13.00^ab^ | 55.56(48.21) ^b^ | 18.78 | 4182.78(64.57) ^ef^ |
| \| **SD (PF)** \| \| --- \| | 75.40^abc^ | 14.60^ab^ | 46.42(42.93) ^bcde^ | 32.13 | 4398.89(66.32) ^bcde^ |
| \| **ST+SD (PF)** \| \| --- \| | 70.27^bc^ | 13.80^ab^ | 50.25(45.14) ^bcd^ | 26.54 | 4335.00(65.82) ^bcde^ |
| \| **ST+SD+FS(PF)** \| \| --- \| | 82.20^ab^ | 14.87^ab^ | 40.00(39.15) ^cde^ | 41.52 | 4858.89(69.71) ^abcd^ |
| \| **Hexaconazole(5SC)** \| \| --- \| | 83.33^a^ | 14.20^ab^ | 36.30(36.99) ^e^ | 46.94 | 5098.89(71.41) ^a^ |
| \| **Control** \| \| --- \| | 67.00^c^ | 11.67^ab^ | 68.40(56.14) ^a^ | - | 3626.67(60.22) ^f^ |

PF- Pseudomonas *fluorescence; **values in parentheses in PDI are arcsine-transformed values. ** Values in parentheses in yield are square root transformed values. PDI, percent disease index; DAT, days after transplanting; PDOC, percent disease over control. Means in a column followed by same letters are not significantly different according to DMRT at 5% level.
